# The changes in socioeconomic inequalities and inequities in health services utilization among patients with hypertension in Pearl River Delta of China, 2015 and 2019

**DOI:** 10.1186/s12889-021-10879-6

**Published:** 2021-05-12

**Authors:** Yan Liu, Nan Liu, Mengjiao Cheng, Xin Peng, Junxuan Huang, Jinxiang Ma, Peixi Wang

**Affiliations:** 1 Institute of Chronic Disease Risks Assessment, Henan University, Jinming Campus, Kaifeng, Henan China; 2 School of Public Health, Guangzhou Medical University, Guangzhou, Guangdong China

**Keywords:** Health service utilization, Hypertension, Socioeconomic inequality, Horizontal inequity, China

## Abstract

**Background:**

Assessing inequities in health services utilization contributes to build effective strategies for health equity promotion. This study aimed to evaluate the socioeconomic inequalities and inequities in health services utilization among hypertensive patients and explore the changes between 2015 and 2019 in Pearl River Delta of China.

**Methods:**

The cross-sectional surveys were conducted using the questionnaire. Eight hundred thirty and one thousand one hundred sixty-six hypertensive patients in 2015 and 2019 were interviewed, respectively. The concentration index (CI) and the horizontal inequity index (HI) were used to access the socioeconomic inequalities and horizontal inequities in outpatient and inpatient health services use. The contribution of influential factors to the overall inequalities was estimated via the concentration index decomposition. Oaxaca-type decomposition technique was utilized to measure the changes in socioeconomic inequalities between the observation periods.

**Results:**

In 2015 and 2019, the CIs for outpatient and inpatient utilization decreased from 0.1498 to 0.1198, 0.1982 to 0.1648, respectively, and the HIs for outpatient and inpatient utilization decreased from 0.1478 to 0.1078, 0.1956 to 0.1390, respectively. Economic status contributed the maximum ratio of the socioeconomic inequalities in the use of outpatient service (81.05% in 2015, 112.89% in 2019) and inpatient service (82.46% in 2015, 114.68% in 2019) in these 2 years. Oaxaca decomposition revealed that educational level (78.30% in outpatient, 53.79% in inpatient) and time to the nearest health facilities (66.78% in outpatient, 31.06% in inpatient) made the main positive contributions to decline the inequalities. While the main factor pushing the equalities toward deterioration was economic status (− 46.11% in outpatient, −76.56% in inpatient).

**Conclusion:**

There were certain declines in the socioeconomic inequalities and inequities in health services utilization by hypertensive patients in Pearl River Delta of China over time. The widening economic gap was the largest contribution to the observed inequalities. Interventions to protect the vulnerable group deserve further concern from policy makers.

**Supplementary Information:**

The online version contains supplementary material available at 10.1186/s12889-021-10879-6.

## Background

In the past few decades, reducing inequity in the utilization of health care has been a major goal of health systems [1]. However, it is still being observed globally [2–4], even exists in nations that are considered as “more equitable”, such as Norway [5]. China, as the greatest developing country, is no exception. In the report issued by the World Health Organization in 2000, China ranked 188th out of 191 countries in health performance [6].

Indeed, since China began its economic reform in 1978, the gap between the rich and poor has widened and health insurance coverage was small. The sick had to pay a high medical expense, while the poor could not support themselves due to financial difficulty [7]. Fortunately, the seriousness of the issue has drawn the attentions of the Chinese government, and the government had carried out some relevant policies, such as the launch of a new health system reform in 2009. Measures to promote a more equitable health system were implemented in the reform, including strengthening the primary health care system, expanding health insurance coverage, and subsidizing the funding of public health services in poor areas [8]. The number of practicing (assistant) physicians per thousand population increased by 65% from 2009 (1.7) to 2019 (2.8) [9], which was one result of this reform. Universal health coverage is another outstanding achievement. The coverage increased from 56% in urban and 21% in rural in 2003 to almost 90% in 2015 [10, 11]. However, there are strong pieces of evidence of pro-rich inequities in the utilization of health services [12, 13]. Equity of access to health service in the Chinese health care system still a serious concern.

Previous studies have been carried out worldwide on the inequity in the utilization of health services. A study conducted in Norway showed that low-income and less educated individuals as a vulnerable group in the process of seeking hospital outpatient care [5]. Another report observed that the utilization of health care showed a pro-rich inequity in the slum areas, whereas horizontal equity was achieved among the non-slum areas [14]. Terraneo found that compared with the less educated elderly, the more educated make more use of health services because they have more resources, such as cognition, communication, and relationships, which enable them to make more informed choices [15]. It also showed that the poor is facing worse health situations and they require more urgent health services than the rich [16]. Barbosa used the multivariate inequity to analyze the utilization of physician service in Brazil, and found that health insurance coverage and urban location both contribute more to the pro-rich inequity than income [17].

At present, the inequity in health services utilization studies in China are mainly focused in a specific area such as the rural region [13], and special communities such as the middle-aged and elderly [18]. However, there is still a lack of comparative studies on the inequities of outpatient and inpatient services for individuals with chronic non-communicable diseases (NCDs) [19], especially among those with hypertension. Hypertension was ranked the 1st in the number of two-week illnesses and chronic diseases in China according to the 2013 National Health Services Survey [20], and 6.6% of health-care costs are directly related to hypertension [21]. More seriously, compared with other diseases, patients with hypertension often require more frequencies and long-term connecting to health facilities. It has been considered as one significant financial burden to the lower socioeconomic groups which tend to use fewer health services, and it will exacerbate the inequities in the utilization of health services. Therefore, it is important and urgent to pay close attention to the socioeconomic inequality and horizontal inequity of outpatient and inpatient services utilization in patients with hypertension.

The Pearl River Delta, as a major part of Guangdong-Hong Kong-Macao Great Bay Area, is one of the most open and economically dynamic regions in China. At the same time, the medical and health services system in this region is booming. For example, the number of health care institutions increased by 14% from 2009 (17,433) to 2015 (19,870). Of note, the number increased to 25,023 in 2019, which was up 26% from 2015 [22]. However, this area has a heavy burden on NCDs. It is noted that more than one-fifth of middle-aged and elderly residents suffer from two or more chronic diseases [23]. As the development of this region, it attracts not only many domestic migrants from other provinces, but also residents from Hong Kong and Macao. Recent policy has proposed that residents of Hong Kong and Macao who living in Guangdong Province could share the same treatment as mainland residents in education, medical care and old-age care, etc. [24]. This new requirement means that cooperation between the region will no longer be restricted in the economy, but will also focus on society and livelihood issues. A society should grant its citizens equal access to health care for equal need. Under the special circumstances of “one country, two systems”, it is especially crucial to explore the inequities in the utilization of health services and promote the harmonious development of the Great Bay Area.

In such context, this study is to measure socioeconomic inequalities and inequities in health services utilization by hypertensive patients in Pearl River Delta in 2015 and 2019 and to explore whether the condition have been changed. Using decomposition analysis, this study also provides further evidence on the contribution of socioeconomic variables to the distribution of health services utilization among hypertensive individuals.

## Methods

### Study design and subjects

A data from the cross-sectional study based on a community health survey conducted in Pearl River Delta of China in 2019 was used. Moreover, the data from the same survey we conducted in this region in 2015 was used for comparison. The two study samples were selected by a multistage and stratified random sampling method. The primary sampling units were designated as street communities and the second-stage sampling units were nominated as communities according to the economic level. All information was collected by trained staff through face-to-face household questionnaire interviews, including demographics, socioeconomic variables, and health services utilization. A total of 6153 and 6426 individuals aged 15 and above were sampled from 138 communities or villages in 2015 and 2019, respectively. After excluding data with relevant variables missing and logic error answers, complete data were available for 5507 individuals in 2015, and 5867 individuals in 2019. Overall, 830 and 1166 residents suffered from hypertension in 2015 (15.1%) and 2019 (19.9%) were selected for further analysis.

### Measurements

#### Dependent variables

Two binary outcome variables of health services utilization by hypertensive patients were employed. The following questions of the hypertensive patients were asked: (1) Have you visited a doctor for outpatient care because of hypertension in the past 2 weeks? (2) Have you visited a doctor for inpatient care as a result of hypertension in the past year? The answers to the questions were either “yes” or “no”.

#### Independent and control variables

In our study, age, sex, and years of hypertension were classified as the need variables. Age was categorized into three-year groups within 65 years old and years of hypertension was also divided into three groups: 1–4, 5–9, and 10 or above.

Economic status, educational level, employment status, marital status, residential location, registration, health insurance, and time to the nearest health facilities were considered as socioeconomic variables (non-need variables).

Household income can directly summarize an individual’s social position and possession of resources that are important for health [25], so we measured economic status by the per capita household income, which was calculated by dividing the total income last year by household size. Total income refers to the income of the household for final expenditure and savings, including cash receipts and in-kind receipts. Then we divided per capita household income into five quintiles, which meant that the lowest and the highest quintile represent the poorest and the richest wealthy quintiles, respectively. In this study, the per capita house income data were inflated to 2019. Finally, the per capita household income in the participants in 2015 was grouped as follows: ≤8738 RMB; 8739 ~ 18,022 RMB; 18,023 ~ 26,947 RMB; 26,948 ~ 34,909 RMB and ≥ 34,910 RMB. The per capita household income in the participants in 2019 was ordered as follows: ≤11,484 RMB; 11,485 ~ 23,760 RMB; 23,761 ~ 40,764 RMB; 40,765 ~ 55,932 RMB and ≥ 55,933 RMB.

Educational levels were grouped into four groups: primary or below education, middle school, high school, and college or above education. Two employment categories and marital categories were employment and unemployment, married and single, respectively. The single includes unmarried, divorced, widowed and separated. Residential location was classified as rural and urban depending on whether the participant resided in an administrative community or village within the last 6 months. The registration was divided into migrants and locals according to whether their domicile is in Guangdong province. Health insurance is based on whether the respondent is covered by the social health insurances. The time to get to the nearest health facilities as fast as possible was divided into two groups: ≤15 min and > 15 min.

#### Concentration index (CI)

The measurement of socioeconomic inequality in the use of health services is based on a widely accepted index, appointed as CI. CI ranges from − 1 to 1, with the positive value indicating the concentration of the health inequality among the rich and vice versa [26]. The index can be calculated by employing the equation as follows, and the definition of each variable in eq. (1) has been described elsewhere [27].

1 $$ C=\frac{2}{\mu}\mathrm{co}\nu \left({y}_i,{r}_i\right) $$

#### Decomposition of inequality

To analyze the contribution of independent variables of the inequality, we followed the method proposed by Wagstaff et al. to decompose CI [28]. Probit regressions were employed to calculate the partial effects [27]. The outcome variable (y) is established as the eq. (2):

2 $$ {y}_i={\alpha}_j^m+{\sum}_j{\beta}_j^m{x}_{ji}+{\sum}_k{\gamma}_k^m{z}_{ki}+{\varepsilon}_i $$

In eq. (2), where *x*_*ji*_ is the need variable (e.g. age, sex, and health need), *z*_*ki*_ is the non-need or socioeconomic variable (e.g. economic status, educational level, and employment status); *β*_*j*_ and *γ*_*k*_ are the marginal effects (*dy*/*dx*) of *x*_*j*_ and *z*_*k*_; *ε*_*i*_ indicates the error term. Then, CI for y can be calculated as the eq. (3):

3 $$ C={\sum}_j\frac{\beta_j^m\overline{x_j}}{\mu }{C}_j+{\sum}_k\frac{\gamma_k^m\overline{z_k}}{\mu }{C}_k+\frac{G{C}_{\varepsilon }}{\mu } $$

In eq. (3), where $\overline{\chi_{\mathrm{j}}}$ and $\overline{Z_k}$ are the means of *x*_*j*_ and *z*_*k*_; *C*_*j*_ and *C*_*k*_ are the concentration indexes of *x*_*j*_ and *z*_*k*_; *μ* is the mean of y and GC_*ε*_ is the concentration index of the error term ε. This equation reveals the total concentration index is made up of two components. One of them is the residual, another one is the determinant part [29]. Then we calculated the absolute and the percentage contribution of each regressor. Details of the contribution have been described elsewhere [27].

#### Horizontal inequity index (HI)

HI is the concentration index that measures the need-standardized health service utilization. It reflects socioeconomic inequality in the use of health services after controlling for the impacts of biological needs, such as age and sex [30]. HI can be computed as follows:

4 $$ \mathrm{HI}=\mathrm{C}\hbox{-} {\sum}_j\left(\frac{\beta_j^m\overline{x_j}}{\mu}\right)\kern0.5em {C}_j $$

In eq. (4), similar to CI, HI ranges from − 1 to 1. A positive HI suggests that the health service is more concentrated among the richer groups and vice versa.

#### Decomposition changes in inequality

At this stage, the Oaxaca-type decomposition method was used to decompose the change in inequality [31]. The decomposition formula is as follows and the details of the definition of each variable in eq. (5) have been described elsewhere [32].

5 $$ \varDelta \mathrm{C}={\sum}_k{\eta}_{kt}\left({c}_{kt}-{c}_{kt-1}\right)+{\sum}_k{c}_{kt-1}\left({\eta}_{kt}-{\eta}_{kt-1}\right)+\varDelta \frac{GC_{\varepsilon t}}{\mu_t} $$

All analyses were performed on STATA 14.0. Statistical significance level was set as 0.05.

## Results

### Social demographic characteristics of respondents

The characteristics of the study population are displayed in Table 1. In 2015 and 2019, the utilization of inpatient service due to hypertension increased greatly, with the growth rate at 34.20%, whereas, a slight rise in the utilization of outpatient service, with a growth rate of 10.64%. Nearly half of the patients were aged 65 or above. More than half of the patients were suffering from hypertension for less than 5 years. Most of the respondents finished middle school. Over half of the participants were unemployed and married. Most of the respondents with hypertension were urban and local residents. The health insurance coverage increased from 97.71 to 99.94% during this period. In terms of access to care, it took most of them less than 15 min to get to the nearest health facilities.

**Table 1:** Characteristics of study participants

Variables	2015(*n* = 830)		2019(*n* = 1166)	
	N	%	N	%
Dependent variables				
Outpatient utilization				
No	610	73.49	824	70.67
Yes	220	26.51	342	29.33
Inpatient utilization				
No	690	83.13	902	77.36
Yes	140	16.87	264	22.64
Need variables				
Age(y)				
15–44	79	9.52	134	11.49
45–54	117	14.10	184	15.78
55–64	224	26.99	296	25.39
≧65	410	49.40	552	47.34
Sex				
Female	436	52.53	619	53.09
Male	394	47.47	547	46.91
Years of hypertension (y)				
0–4	467	56.27	665	57.03
5–9	209	25.18	323	27.70
≧10	154	18.55	178	15.27
Socioeconomic variables				
Economic status				
Poorest	169	20.36	233	19.98
Poorer	170	20.48	235	20.15
Middle	165	19.88	234	20.07
Richer	165	19.88	233	19.98
Richest	161	19.40	231	19.81
Educational level				
Primary or below	372	44.82	529	45.37
Middle school	257	30.96	358	30.70
High school	146	17.59	198	16.98
College or above	55	6.63	81	6.95
Employment status				
Unemployed	555	66.87	723	62.01
Employed	275	33.13	443	37.99
Marital status				
Single	137	16.51	180	15.44
Married	693	83.49	986	84.56
Residential location				
Rural	323	38.92	511	43.83
Urban	507	61.08	655	56.17
Registration				
Migrants	214	25.78	295	25.30
Locals	616	74.22	871	74.70
Health insurance				
No	19	2.29	7	0.60
Yes	811	97.71	1159	99.40
Time to the nearest health facilities (min)				
≦15	707	85.18	1082	92.80
> 15	123	14.82	84	7.20
CI of outpatient utilization	0.1498		0.1198	
HI of outpatient utilization	0.1478		0.1078	
CI of inpatient utilization	0.1982		0.1648	
HI of inpatient utilization	0.1956		0.1390	

*Note*: *CI* Concentration index, *HI* Horizontal inequity index

### Socioeconomic inequalities and inequities

**Fig. 1:**
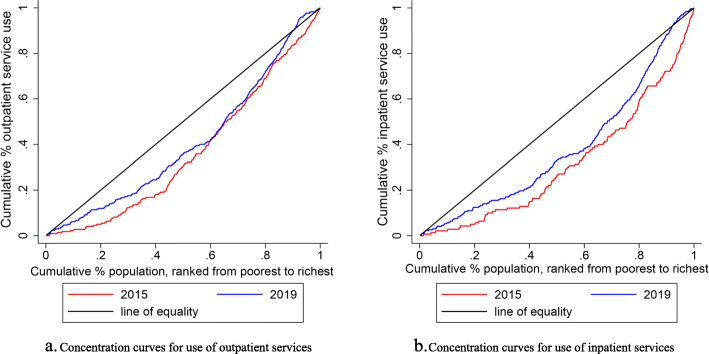
Concentration curves for use of health services, 2015 versus 2019. **a**. Concentration curves for use of outpatient services. **b**. Concentration curves for use of inpatient services

Table 1 also presents CIs and HIs for health services utilization by hypertensive patients. The CIs for outpatient and inpatient utilization, although all of them were positive, decreased from 0.1498 to 0.1198, 0.1982 to 0.1648, respectively, which meant that there were existed pro-rich inequalities in utilization of health services by hypertensive patients, but the inequalities shrank over time (Fig.1). However, as the health services requirement have not been taken into account, inequality is not equivalent to real inequity [18]. Then we calculated the HI. The HIs for outpatient and inpatient utilization decreased from 0.1478 to 0.1078, 0.1956 to 0.1390, respectively, which provides evidence of pro-rich inequities in utilization of health services. In other words, the rich could utilize more health services than the poor even after controlling for their different demands. Compared with the inequities in outpatient utilization, those in inpatient utilization were higher during 2015–2019.

### Decomposition of the inequalities

A positive contribution to socioeconomic inequality means that the considered variable increases inequality. Tables 2 and 3 reported the detailed decomposition of CIs for the health services utilization by hypertensive patients in the two survey-years. As can be seen from the results, the need variables of 65 years or above and years of hypertension more than 10 years displayed contribution in favor of the affluent. Among socioeconomic variables, economic status played the greatest contributory role to the inequalities favoring the rich in the utilization of health services.

**Table 2:** Decomposition of CI in outpatient utilization by hypertensive patients in 2015 and 2019

Variables	2015(*n* = 830)			2019(*n* = 1166)		
	*dy*/*dx*	Con.	%con.	*dy*/*dx*	Con.	%con.
Age(y)						
15–44	Ref.					
45–54	0.1017	−0.0023	−1.55	− 0.0442	0.0012	0.99
55–64	0.1081	0.0018	1.23	0.0947	− 0.0004	− 0.30
≧65	0.1873*	0.0016	1.07	0.1715*	0.0122	10.21
sum		0.0011	0.75		0.0131	10.90
Sex						
Female	Ref.					
Male	− 0.0329	− 0.0013	− 0.84	− 0.0001	< 0.0001	< 0.01
Years of hypertension (y)						
1–4	Ref.					
5–9	0.2398*	−0.0006	−0.39	0.0548	−0.0012	−1.03
≧10	0.1583*	0.0026	1.72	0.0866*	0.0038	3.21
sum		0.0020	1.33		0.0026	2.19
Economic status						
Poorest	Ref.					
Poorer	0.0450	−0.0068	−4.53	0.0699	−0.0096	−7.98
Middle	0.2651*	0.0010	0.67	0.1841*	0.0001	0.11
Richer	0.3294*	0.0507	33.86	0.3796*	0.0520	43.43
Richest	0.2577*	0.0765	51.05	0.3418*	0.0926	77.34
sum		0.1214	81.05		0.1352	112.89
Educational level						
Primary or below	Ref.					
Middle school	0.0182	−0.0003	−0.19	0.0495	−0.0017	−1.41
High school	0.1224*	0.0055	3.69	0.2329*	0.0003	0.23
College or above	0.3888*	0.0157	10.49	0.2243*	−0.0011	−0.94
sum		0.0210	13.99		−0.0025	−2.12
Employment status						
Unemployed	Ref.					
Employed	0.0002	< 0.0001	−0.01	0.0786	−0.0051	−4.29
Marital status						
Single	Ref.					
Married	0.0146	−0.0002	− 0.14	0.0031	< 0.0001	−0.03
Residential location						
Rural	Ref.					
Urban	0.0756*	0.0056	3.76	0.0298	0.0010	0.80
Registration						
Migrants	Ref.					
Locals	0.1424*	0.0158	10.52	0.2446*	0.0051	4.24
Health insurance						
No	Ref.					
Yes	0.0932	0.0006	0.37	0.0116	< 0.0001	0.02
Time to the nearest health facilities (min)						
≦15	Ref.					
> 15	−0.2265*	0.0216	14.43	−0.2342*	0.0016	1.32

Note: (1) *dy*/*dx*: Partial effect in probit regression model; Con.: The absolute contribution of each determinant; %con.: The percentage contribution of each determinant to the total concentration index; (2) *: *P* < 0.05

**Table 3:** Decomposition of CI in inpatient utilization by hypertensive patients in 2015–2019

Variables	2015(n = 830)			2019(n = 1166)		
	*dy*/*dx*	Con.	%con.	*dy*/*dx*	Con.	%con.
Age(y)						
15–44	Ref.					
45–54	0.1159*	−0.0042	−2.10	− 0.0030	0.0001	0.06
55–64	0.0753	0.0020	1.01	0.0987	−0.0005	− 0.29
≧65	0.1323*	0.0018	0.89	0.1700*	0.0157	9.53
sum		−0.0004	− 0.19		0.0153	9.30
Sex						
Female	Ref.					
Male	− 0.0234	− 0.0014	− 0.71	0.0424	− 0.0016	−1.00
Years of hypertension (y)						
1–4	Ref.					
5–9	0.1702*	−0.0007	−0.33	0.0225	−0.0007	− 0.40
≧10	0.2387*	0.0061	3.08	0.2009*	0.0116	7.02
sum		0.0055	2.75		0.0109	6.62
Economic status						
Poorest	Ref.					
Poorer	−0.0109	0.0026	1.30	0.0078	−0.0014	−0.84
Middle	0.1372*	0.0008	0.41	0.1916*	0.0002	0.11
Richer	0.1952*	0.0472	23.84	0.3159*	0.0561	34.04
Richest	0.2418*	0.1128	56.91	0.3818*	0.1341	81.38
sum		0.1634	82.46		0.1890	114.68
Educational level						
Primary or below	Ref.					
Middle school	0.0182	− 0.0005	− 0.23	0.0181	− 0.0008	− 0.49
High school	0.0818*	0.0058	2.94	0.0989*	0.0002	0.09
College or above	0.1790*	0.0114	5.73	0.0913	−0.0006	−0.36
sum		0.0167	8.44		−0.0012	−0.75
Employment status						
Unemployed	Ref.					
Employed	0.0427	−0.0006	− 0.28	0.0308	− 0.0026	−1.58
Marital status						
Single	Ref.					
Married	−0.0031	0.0001	0.03	0.0387	−0.0006	−0.34
Residential location						
Rural	Ref.					
Urban	0.0274	0.0032	1.62	0.0363	0.0015	0.92
Registration						
Migrants	Ref.					
Locals	0.1007*	0.0175	8.83	0.1237*	0.0033	2.02
Health insurance						
No	Ref.					
Yes	0.0225	0.0002	0.11	−0.0181	< 0.0001	−0.02
Time to the nearest health facilities (min)						
≦15	Ref.					
> 15	−0.0732*	0.0110	5.54	−0.0690	0.0006	0.37

Note: (1): Partial effect in probit regression mode; Con.: The absolute contribution of each determinant; %con.: The percentage contribution of each determinant to the total concentration index; (2) *: *P* < 0.05

Notably, the percentage contribution of educational level to the uneven distribution of health services was positive in 2015. Nonetheless, it was negative in 2019, especially in hypertensive patients who completed college or above. In other words, the change of this factor reduced such pro-rich inequalities considerably. Furthermore, the positive contributions of the factors such as registration and time to the nearest health facilities declined to varying degrees over time, which also reduced the observed inequalities. For other remained variables such as employment status, residential location and health insurance, their contributions were relatively small during the observation period.

### Decomposition changes in inequalities between 2015 and 2019

As shown in Table 1, the CIs of outpatient and inpatient utilization by hypertensive patients reduced by 0.0300(20.03%) and 0.0334 (16.85%) in 2015 and 2019. Then, the reductions were decomposed to seek contributing factors following by Oaxaca-type decomposition in Table 4. It displayed changes in the amount of inequality in determinants in the second and fourth columns; and the third and seventh columns show changes in elasticities of determinants.

**Table 4:** Oaxaca-type decomposition for changes in inequalities in health services utilization by hypertensive patients, 2015–2019

Variables	Outpatient utilization				Inpatient utilization			
	Δ*c* ∗ *η*_*kt* − 1_	Δη∗ c_kt_ − 1	Change	%	Δc ∗ η_kt_ − 1	Δη∗ c_kt_ − 1	Change	%
Age(y)								
15–44	Ref.							
45–54	0.0002	0.0033	0.0035	−11.69	< 0.0001	0.0042	0.0043	−12.75
55–64	−0.0017	−0.0005	− 0.0022	7.32	− 0.0023	−0.0002	− 0.0025	7.47
≧65	0.0110	−0.0003	0.0106	−35.44	0.0141	−0.0001	0.0139	−41.71
sum				−39.82				−46.99
Sex								
Female	Ref.							
Male	< 0.0001	0.0013	0.0013	−4.20	−0.0035	0.0033	−0.0002	0.73
Years of hypertension (y)								
1–4	Ref.							
5–9	−0.0011	0.0005	−0.0006	2.13	−0.0006	0.0006	< 0.0001	−0.01
≧10	0.0028	−0.0015	0.0013	−4.23	0.0084	−0.0030	0.0055	−16.33
sum				−2.10				−16.34
Economic status								
Poorest	Ref.							
Poorer	−0.0002	−0.0026	−0.0028	9.25	< 0.0001	−0.0039	−0.0040	11.86
Middle	−0.0005	−0.0004	− 0.0009	2.92	− 0.0007	< 0.0001	− 0.0006	1.93
Richer	−0.0011	0.0024	0.0013	−4.37	−0.0011	0.0100	0.0089	−26.51
Richest	−0.0010	0.0171	0.0162	−53.92	−0.0014	0.0227	0.0213	−63.84
sum				−46.11				−76.56
Educational level								
Primary or below	Ref.							
Middle school	−0.0010	−0.0004	−0.0014	4.65	−0.0005	0.0001	−0.0003	1.03
High school	−0.0089	0.0037	−0.0053	17.53	−0.0049	−0.0008	− 0.0057	16.96
College or above	−0.0097	−0.0071	− 0.0168	56.12	− 0.0051	−0.0068	− 0.0120	35.80
sum				78.30				53.79
Employment status								
Unemployed	Ref.							
Employed	0.0016	−0.0067	−0.0051	17.08	−0.0023	0.0002	−0.0021	6.15
Marital status								
Single	Ref.							
Married	< 0.0001	0.0002	0.0002	−0.57	0.0001	−0.0007	− 0.0006	1.87
Residential location								
Rural	Ref.							
Urban	−0.0009	−0.0038	−0.0047	15.55	−0.0014	− 0.0003	−0.0017	5.03
Registration								
Migrants	Ref.							
Locals	−0.0195	0.0089	−0.0107	35.59	−0.0128	− 0.0014	−0.0142	42.45
Health insurance								
No	Ref.							
Yes	< 0.0001	−0.0005	−0.0005	1.78	0.0001	−0.0003	−0.0003	0.75
Time to the nearest health facilities (min)								
≧15	Ref.							
> 15	−0.0082	−0.0118	−0.0200	66.78	−0.0031	− 0.0072	−0.0104	31.06

Note: Δ*c* ∗ *η*_*kt* − 1_: Change in the amount of inequality in determinants; Δη∗ c_kt_ − 1: Change in elasticities of determinants; Change: Change of each determinant; %: Percentage change of each determinant to the change in total inequality

From the changed CIs and elasticities for utilization of health services, we found that the three major contributors to reducing the decrease inequalities, including the educational level, registration, and time to the nearest health facilities. What’s more, changes in employment status, residential location, and health insurance could explain the reduction of CIs to some extent.

However, the changes in economic status accounted for the biggest contributor to the pro-rich inequalities. Also, the need variables of age and years of hypertension pushed these inequalities into deterioration, especially in the utilization of inpatient.

## Discussion

Our study explored the socioeconomic inequalities and inequities in the health services utilization by hypertensive patients in Pearl River Delta of China between 2015 and 2019, and further quantifies the contribution of selected factors toward the inequalities. Besides, we also assessed the changes in the inequalities and inequities during the survey-period. The main findings were as follows: 1) Obvious pro-rich inequalities and inequities in utilization of health services by hypertensive patients existed in Pearl River Delta in both periods, but they declined over time. 2) The changes in such inequalities were caused by the alteration in the interaction among the relevant determinants, including economic status, educational level, employment status, residential location, registration, health insurance and time to the nearest health facilities.

The horizontal inequity indexes in 2015 and 2019 showed that there existed pro-rich inequities in utilization of health services by hypertensive patients in Pearl River Delta, which indicated that more health resources were utilized by the wealthier even after controlling people’s different needs. These findings were consistent with the previous studies [33, 34]. However, the study displayed an exciting sign that the degrees of inequities in the use of health services have decreased over time. It could be explained by the measures taken by the Chinese government to ensure equity to health access [35, 36]. The funding subsidy for basic public health services increased from 15 to 40 RMB per capita from 2009 to 2015, even increased to 69 RMB per capita in 2019 [37–39]. For hypertensive patients, the Chinese government provides at least one free follow-up visit every 3 months including health evaluation, syndrome surveillance, behavioral intervention, guidance on the use of medicines, and health education [40]. Naturally, these initiatives are supposed to help reduce the disease burden and improve the equities in health services utilization.

In line with other studies [16, 41], HIs also revealed greater inequities in inpatient utilization compared to outpatient utilization in both 2015 and 2019. As the national report showed, financial difficulty is the main reason why low-income people refuse or avoid hospitalization [20]. The price of inpatient service may be much more expensive than the outpatient, and hypertension patients from lower economic status are easier to fall into poverty [16].What’s worse, many low-income people are located in rural areas, which have poor access to hospitals [42]. Compared with the urban patients, rural patients will incur additional costs during hospitalization, such as accommodation and transportation costs, which greatly increase the indirect hospitalization costs. They are more likely to enter a vicious circle of “poverty from illness and disease from poverty”. Besides, the reimbursement rate of hypertensive patients attending basic medical insurance is as high as more than 50%, when they use outpatient services in secondary and lower designated primary medical institutions [43],with no doubt that low-income patients are more likely to choose the outpatient service at an affordable cost than inpatient service by Chinese medical service policy.

Previous literature showed that the pro-rich inequity in the inpatient service in Canada was decreasing over time, while such inequity in outpatient was increasing [1]. However, our survey indicated that the pro-rich inequities in inpatient and outpatient services in the Pearl River Delta of China were gradually reduced over time. Based on an ongoing nationally longitudinal data in China by Xu’s report, it also showed that the inequity of preventive service use in China was alleviating [32]. Findings from some developing countries, such as Mongolia and Mexico [3, 44], indicated the same trend in the inequity in health service utility as ours. Brazil was a bit different, where inpatient service was always in favor of the poor between 1998 and 2008, although the inequity was declining [4]. Affordability and accessibility are the two main components of equal access to qualified health services. The affordability is primarily related to health insurance reimbursement rates and income-related factors; and the availability is primarily related to the allocation of health resources and government policies [42]. As we can see, so great efforts on the two components were made by the Chinese government in the past few years, that the inequalities from 2015 to 2019 were gradually reduced. Notably, despite the inequity in the inpatient service in the Pearl River Delta has decreased in 2019, HI was still higher than that of Mongolia and Mexico [3, 44]. It follows that horizontal equity in health care remains a challenge for this region of China and the government still needs to focus more on this issue.

The decomposition analysis presented that economic status had the most significant association with inequalities in the use of health services by hypertensive patients in Pearl River Delta of China during 2015–2019, furthermore, the positive contribution was increased in 2019. Actually, many studies have proved that the economic status was associated with health services utilization owing to high payment capacity for care by high incomes [45, 46]. It also found that the rich with NCDs tended to overuse the health services, in contrast, the poor with NCDs tended to underuse the health services [41]. However, as Fu’s study showed, people in lower income are generally in poorer health and they have a greater demand for health services [12]. Clearly, the contribution of the economic status was not conducive to the equitable and rational utilization of health services by individuals.

In addition, other socioeconomic variables such as educational level, employment status, residential location, registration and time to the nearest health facilities also can help to explain the uneven distribution of health services utilization over this period [42, 47–49]. Generally, a variety of social factors related to an individual’s socioeconomic status have an impact on a person’s health beliefs, which in turn influence one’s health-seeking behavior [48].

Oaxaca decomposition revealed that the changes in inequalities arise from the alteration in the interaction among the related determinants, including economic status, educational level, employment status, residential location, registration, health insurance and time to the nearest health facilities. Changes in the residential location, registration, and time to the nearest health facilities pushed the inequalities towards equality line. These findings were likely to reflect the successful outcome of China’s new health reform and the implementation of recent policies, which aimed at establishing a public health system covering both urban and rural residents and a universal health insurance system through affordable and equitable primary health care [50]. The number of health facilities nationwide reached 1.014 million in 2019, an increase of 24,000 over 2015 [11, 51], even hypertensive patients living in townships and counties can easily get a variety of antihypertensive drugs at a zero profit mark-up from primary care facilities [52]. A “15-minute medical treatment circle” has basically been formed, greatly reducing the distance and time for residents to obtain health services [53]. Significantly, the government of Guangdong Province has taken the lead in implementing the project of a direct settlement of out-of-town medical treatment to ensure the migrants can obtain timely reimbursement [54]. These measures greatly reduced the burden of the migrants suffered from hypertension and bridged urban-rural disparity in health services utilization.

Moreover, the development of employment status and educational attainment have also contributed to reducing there inequalities. A series of employment policies delivered by the government, such as increasing employment opportunities among the economically disadvantaged groups [55], played a role in promoting this trend. For the changes in educational level, a possible explanation may be that the proportion of young hypertensive individuals substantially increased in China [56], leading to an overall increase in patients’ education. It is well-known that health insurance can help to reduce the inequality in health services use [57, 58]. Our study also showed the same result, but the contribution was really limited. The insurance coverage in China exceeded 90% in 2015 and reached 96% in 2019 [11, 51], and the change in the coverage is only about 6% which may explain why health insurance did not contribute largely.

However, we found that the change in economic status has pushed the inequalities in health services usage towards deterioration, which is consistent with another study in China [32]. It may be related to the growing income gap in China. According to the lasted national data, China’s gross domestic product (GDP) per capita increased from 50,237 yuan in 2015 to 70,581 yuan in 2019 [59]. However, rapid growth has brought increased income inequality over the past few years, with the individual income Gini coefficient rising from 0.462 in 2015 to 0.465 in 2019 [60]. There is no doubt that the inequality of income in China ultimately widened the gap in purchasing healthcare services between the rich and poor.

Also, our findings revealed that changes in the need variable of age and years of hypertension made negative contributions to the reduction in such inequalities, particularly in the utilization of inpatient. High blood pressure can reduce the elasticity of the vascular wall and aggravate atherosclerosis with the development of the disease, causing a variety of heart, brain, kidney and other target organ damage. Hypertensive patients who are older or had a long year of hypertension may have high demands for health services, especially for inpatient service. Thereby, more health services were biased toward those people.

This study has some limitations that must be mentioned. Firstly, recall biases could not be avoided in questionnaire-based surveys, especially the self-reported utilization of health services. Secondly, the supply-side variables used in the decomposition only include the time accessibility of health facilities, but lack of other factors, such as the price of health services. Finally, since the decomposition analysis is a descriptive statistic, we were not able to carry out a causality analysis and the results should be interpreted with care. Despite the above limitations, this study has important policy implications for China towards reducing socioeconomic inequalities and inequities in health services utilization among patients suffered from NCDs.

## Conclusion

Monitoring of the inequities in health services for people with NCDs can help design effective strategies to improve health equity. Overall, our results indicated that the pro-rich inequities persist in health services utilization by hypertensive patients in Pearl River Delta from 2015 to 2019 and the horizontal inequity in the utilization of inpatient service is higher than that of outpatient service, although there is a reduction of the inequities. The Oaxaca decomposition analysis revealed that the widening income gap is the main factor to exacerbate such inequalities. Health policies alone are not enough to tackle the inequities and more comprehensive social policies are needed to protect the disadvantaged groups, particularly the individuals who suffered from chronic diseases.

## Supplementary Information

**Additional file 1: **The questionnaire used in this study. **Table 1.** Questionnaire on Family General Situation (excerpt). **Table 2.** Questionnaire on Personal Situation of Family Members (excerpt).

## Data Availability

The data used during the current study available from the corresponding author (peixi001@163.com) on reasonable request.

## Abbreviations

CI: Concentration index
HI: Horizontal inequity
NCDs: Non-communicable diseases
GDP: Gross domestic product
