# Supplementary material for: The changes in socioeconomic inequalities and inequities in health services utilization among patients with hypertension in Pearl River Delta of China, 2015 and 2019
Source: BMC Public Health. 2021 May 12;21:903. doi: 10.1186/s12889-021-10879-6 (PMC8117279; doi:10.1186/s12889-021-10879-6)
Supplement: Supplementary file 1 — Additional file 1: The questionnaire used in this study. Table 1. Questionnaire on Family General Situation (excerpt). Table 2. Questionnaire on Personal Situation of Family Members (excerpt). [file 12889_2021_10879_MOESM1_ESM.pdf]

Community Health Survey Questionnaire

Home address: __________street __________ village(neighborhood)　　　Detailed address:______________________________________

Name of householder:____________________________________

ID card of householder:□□□□□□ □□□□□□□□ □□□□

Telephone No.: ____________________________________

The purpose of this community health diagnosis has been described and informed consent has been obtained from the surveyed households□　Signature: _____________________________

Survey date: _________Month_________Day_________Year

Level 1 quality control officer (signature): ______________

Verified date: _________Month_________Day _________Year

Level 2 quality control officer (signature): _______________

Verified date: _________Month_________Day _________Year

Hello!We are the investigators of the community diagnostic survey. The main purpose of this survey is to understand the health status of the community residents and the utilization of medical and health services, so as to provide information for the improvement of local health policies and the improvement of the health level of the residents. All the survey content is only used for statistical analysis, and the information of you and your family members will be kept strictly confidential. We hope you can answer the following questions truthfully. Thank you very much for your support and cooperation!

Table 1 Questionnaire on Family General Situation（excerpt）

**No.**	Questions and options	Response
1	How many people are there in your household register？ [If there are more than one household registers, fill in the total number of people in each household register]	
2	What has been the total household income in your home in the past year？ [including cash receipts and in-kind receipts]	
3	How long does it take you to get to the nearest health facilities as fast as possible?	

Table 2 Questionnaire on Personal Situation of Family Members（excerpt）

Code number of the member under investigation		01	02	03	04	05	06
4	Name:						
5	Your relationship with householder: (1)Householder himself (2)Spouse (3)Children (4)Son-in-law/Daughter-in-law (5)Parents (6)Parents-in-law (7)Grandparent (8)Grandchild (9)Brother/sister (10)Domestic help staff (11)Others						
6	How do you answer the following survey questions?						

	(1) Answer yourself (2)Other people answer for you						
7	Have you been diagnosed with hypertension by the doctor?<br>(1)Yes (2)No						
8	How many years have you had the disease?<br>[0.5 years for 6 months, 0.25 years for 3 months]						
9	Have you visited a doctor for outpatient care because of hypertension in the past two weeks?<br>(1)Yes (2)No						
10	Have you visited a doctor for inpatient care as a result of hypertension in the past year?<br>(1)Yes (2)No						
11	Your date of birth is: ______ Year (fill in 4 digits, e.g. 1998)						
12	______Month						
13	Your Sex is：(1)Male (2)Female						
14	Your educational level is: (1)Primary or below school (3)Middle school (4)High school [including technical school or secondary professional school] (7)University specialized school (8)University undergraduate school (9) Postgraduate and above school						
15	Your employment status is: (1)Employed [including flexible employed] (2)Leaving/Retired (3)Student (4)Laid-off (5)Job-waiting						
16	Your marital status is: (1)Unmarried (2)Married (3)Divorced (4)Widowed (5)Separated						
17	Your registration is: (1)Local (2)Migrant						
18	Do you have a health insurance？ (1)Yes (2)No						
